# 73 Associations Between Pre-burn Occupation Type and Employment Outcomes at One Year

**DOI:** 10.1093/jbcr/irac012.076

**Published:** 2022-03-23

**Authors:** Lauren J Shepler, Gretchen J Carrougher, Nicole S Gibran, Karen J Kowalske, Barclay T Stewart, Colleen M Ryan, Jeffrey C Schneider

**Affiliations:** Spaulding Rehabilitation Hospital, Boston, Massachusetts; Department of Surgery, The University of Washington, Seattle, Washington; University of Washington, Wellfleet, Massachusetts; Department of Surgery, The University of Texas Southwestern Medical Center, Dallas- Fort worth, Texas; University of Washington, Seattle, Washington; Harvard Medical School, Boston, Massachusetts; , Massachusetts

## Abstract

**Introduction:**

Reintegration into the workforce after burn injury is an important issue for survivors. In a 2012 systematic review, 28% of burn survivors never returned to any form of employment. Although pre-burn employment status is strongly associated with post-burn employment, there are little data on the role of pre-injury occupation type on workplace reintegration. The aim of this project was to assess the impact of occupation type on employment outcomes after burn injury.

**Methods:**

Data from the National Institute on Disability, Independent Living, and Rehabilitation Research Burn Model System National Longitudinal Database from 2015 to 2021 were used to investigate the association between occupation type and employment outcomes. Occupation type was classified into two groups, Labor and Non-labor, using the U.S. Bureau of Labor Statistics Standard Occupational Classification System. Demographic and clinical data were compared between groups. Mixed regression analyses examined associations between pre-burn occupation type and post-burn employment outcomes (employment at 1 year, days to return to work), controlling for age, gender, race, ethnicity, pre-injury employment, and burn size.

**Results:**

Of the 600 patients who were employed pre-injury, 247 (41%) identified with a non-labor occupation and 353 (59%) with labor occupations. The Labor group was more male (82% vs. 61%) and Hispanic (23% vs. 6%), younger (mean age 42.1 vs. 48.3 years), less educated (high school or less, 25% vs. 11%) and more likely to have been injured at work (28% vs. 14%) compared to the Non-labor group (p< 0.001 for all comparisons). Changes in occupation were seen from pre-injury to post-injury; 16% of working survivors changed from Non-labor to Labor and 13% from Labor to Non-labor occupation types. For those who did return to work after injury, the average time to return to work was greater for Labor compared to the Non-labor group (150 vs 100 days; p=0.003). Additionally, those in the pre-injury Labor group were less likely to be employed at 12 months compared to the Non-labor group (odds ratio = 0.41; p=0.009).

**Conclusions:**

Pre-injury occupation type is associated with employment outcomes after injury. Therefore, occupation type can be used to inform vocational reintegration resources, such as vocational rehabilitation programs, to optimize survivor outcomes.

